# Optimizing PBMC Cryopreservation and Utilization for ImmunoSpot^®^ Analysis of Antigen-Specific Memory B Cells

**DOI:** 10.3390/vaccines13070765

**Published:** 2025-07-19

**Authors:** Noémi Becza, Lingling Yao, Paul V. Lehmann, Greg A. Kirchenbaum

**Affiliations:** Research and Development, Cellular Technology Ltd. (CTL), Shaker Heights, OH 44122, USA; noemi.becza@immunospot.com (N.B.); lingling.yao@immunospot.com (L.Y.); paul.lehmann@immunospot.com (P.V.L.)

**Keywords:** ELISPOT, FluoroSpot, immunological memory, humoral, plasma cell, immune monitoring

## Abstract

**Background:** Measuring frequencies of antigen-specific memory B cells (B_mem_), their immunoglobulin (Ig) class and subclass usage, cross-reactivity, and affinity can provide insights into the efficacy of future antibody responses in case of antigen re-encounter. B cell ImmunoSpot^®^ assays can provide such information; however, like most cell-based tests, they require considerable amounts of blood to be drawn from the donor and this has hindered their inclusion in clinical trials and routine immune diagnostics. **Methods**: We introduce strategies for reducing the cell numbers required to 2–3 million peripheral blood mononuclear cells (PBMCs) per antigen, obtainable from 2–3 mL of blood from healthy adult donors. **Results**: Except when B_mem_ frequencies were very low, we found that testing PBMCs in singlet wells, but in serial dilution, enables as reliable B_mem_ frequency assessments as when testing replicate wells at a single fixed cell number. Additionally, B cell ImmunoSpot^®^ assays can be multiplexed for detecting four Ig classes, or IgG subclasses, simultaneously and without loss of sensitivity. The requirement for low cell numbers and the retention of B cell functionality by cryopreserved PBMCs equivalent to freshly isolated material implies that fewer than the standard 10 million PBMCs per vial can be frozen. This would reduce the number of individuals who could not be tested for B_mem_ due to insufficient availability of PBMCs, a common problem with such assays. **Conclusions**: The predictable need for and recovery of cryopreserved PBMCs facilitates planning of and optimal cell utilization in B cell ImmunoSpot^®^ assays and increases the practical feasibility of extensive B_mem_ characterization in larger cohorts.

## 1. Introduction

Immune monitoring for clinical trials and routine diagnostics primarily relies on detecting antigen-specific antibodies in the serum/plasma of test subjects. Such pre-existing antibodies, however, reflect only the ability of the host to immediately combat an invading antigen. This occurs under optimal conditions through instantaneous neutralization (blockade of receptor-binding domains and/or impairing the antigen’s association with host cells), complement fixation, precipitation through formation of immune complexes, and/or opsonization of the antigen to promote its clearance. Accordingly, preformed serum antibodies constitute the first wall of acquired humoral defense [1]. Importantly, antibodies in serum are relatively short-lived molecules which possess a half-life of ~3 weeks or less when not associated with immunoglobulin (Ig)-binding receptors such as the neonatal Fc receptor (FcRn) [2,3,4,5,6]. Their continued presence in serum therefore depends on ongoing replenishment by antibody-secreting plasma cells. While plasma cells per se can be long-lived [7,8], competition for survival niches located primarily in the bone marrow frequently limits their lifespan [9,10]. Subsequently, antibody levels can decline within months after vaccinations or infections [11,12,13], leaving the host susceptible to (re-)infection. Serum antibodies therefore provide fading evidence for past immune encounters and of established immunological memory.

When pre-existing antibodies can no longer prevent (re-)infections, B_mem_ serve as the second wall of acquired humoral defense [1]. B_mem_, in contrast to antibodies in plasma/serum, are long-lived in vivo [14]. While (unlike plasma cells/blasts) B_mem_ do not secrete antibodies constitutively, upon antigen re-encounter they rapidly give rise to secondary immune responses that produce new generations of plasma cells and B_mem_. Having undergone clonal expansion, Ig class switching, and affinity maturation during the previous immune encounter(s), B_mem_-derived secondary antibody responses are faster and more robust compared to the initial antigen encounter. Therefore, studying B_mem_ unveils the immune potential that has been acquired to combat future antigen challenges when pre-existing antibodies are no longer sufficient to convey protection.

When interpreting the frequently discordant results obtained by measuring antigen-specific antibody levels vs. B_mem_ in blood [12], it must be considered that the differentiation of B cells into the plasma cell and B_mem_ lineages follows different instructive pathways (reviewed in [15,16]). During an immune response, antigen-specific (naïve and/or memory) B cells become activated and subsequently are recruited into germinal centers (GCs) within secondary lymphoid organs (spleen and/or lymph nodes) where they undergo additional clonal expansions while also acquiring somatic hypermutations (SHMs) within the genes encoding their B cell antigen receptor (BCR). Subsequently, as a consequence of SHMs, the daughter cells (these are called GC B cells at this differentiation stage) will express BCRs with slightly different affinity for the eliciting antigen. As these SHMs are random, some of the daughter cells will acquire an increased affinity, while others will remain unchanged or even exhibit a reduced affinity for the antigen in comparison to the parental B cell. Through positive selection, the GC B cell subclones with an increased affinity for the eliciting antigen undergo further rounds of proliferation and acquisition of SHMs with only the subclones achieving the highest affinity for the eliciting antigen eventually being recruited into the plasma cell lineage. This process is collectively termed affinity maturation. In contrast, antigen-specific B cells possessing a reduced affinity for the eliciting antigen will exit the germinal center and instead join the B_mem_ cell compartment [16]. As a consequence, the plasma cells (and hence serum antibodies) reflect the high-affinity end of the B cell repertoire generated through the immune response, whereas the B_mem_ repertoire will also include subclones with lower affinity for the antigen. The immunological significance of this dichotomy is that plasma cells predominantly contribute to protection against the original version of an invading infectious organism (i.e., the “homotype”) whereas B_mem_ also prepare the host for encounters with newly emerging future variants (“heterotypes”); while some B_mem_ subclones will possess a modest affinity for the homotype, they may fortuitously be endowed with an increased affinity for a heterotype and enable an anamnestic (secondary) antibody response even at the first encounter with the heterotype [17]. Because of the distinct requirements for plasma cell and B_mem_ lineage differentiation, and the variability in plasma cell life spans, the frequencies of antigen-specific B_mem_ and serum antibody levels are frequently discordant [12] and the former are more reliable in revealing past infections than plasma/serum antibody titers ([12] and Kirchenbaum, manuscript in preparation). Circulating antibodies and B_mem_, reflecting wall 1 vs. wall 2, respectively, reveal different aspects of acquired humoral immunity, the understanding of which is equally important for immune monitoring.

For all the above reasons, the need has emerged to include assessment of antigen-specific B_mem_, encompassing their affinity distribution and cross-reactivity profiles, in immunodiagnostics [18]. Once isolated from the body, serum antibodies are stable and can be stored and shipped at 4 °C (or frozen) for years. Moreover, as they detect abundant molecules in solution, many antibody-detecting tests can be performed with minute quantities of serum. Both of these properties facilitate sero-diagnostics and explain its widespread usage. B_mem_, in contrast, perish shortly after isolation from the body, and either need to be tested right away or cryopreserved for later testing [19,20]. Furthermore, because antigen-specific B_mem_ are rare among peripheral blood mononuclear cells (PBMCs), typically constituting << 0.1% thereof [21], substantial numbers of PBMCs are commonly required for their reliable detection and study. Optimizing PBMC utilization for B_mem_ analysis is therefore critical for the inclusion of these important assays in the immune diagnostic armamentarium; this is the focus of the present communication.

Antigen-specific B_mem_ are best detected via the specificity of the surface BCR they express or, alternatively, based on the specificity of the antibody they secrete following terminal differentiation. One technique suitable for this purpose is staining PBMCs with labeled antigen probes, followed by the identification of antigen-probe-labeled B cells by flow cytometry [21,22]. The other technique relies on detecting antigen-specific B cells via the antibody secretory footprint they generate when seeded onto antigen-coated membranes [23,24]; this test system is called enzyme-linked immunosorbent spot assay (ELISPOT) when the detection of the antigen-bound antibodies occurs through enzyme-catalyzed precipitation and deposition of a visible substrate, or FluoroSpot, if fluorescence-tagged detection antibodies are used. As both assay variants follow the same basic principle and differ only in the means of visualizing the secretory footprints, here they are collectively termed ImmunoSpot^®^. In this communication we focus exclusively on ImmunoSpot^®^ assays because, compared to antigen probe staining, B cell ImmunoSpot^®^ assays require fewer PBMCs, are more suitable for high-throughput work flows, can be automated, and can be readily validated for clinical testing [20].

## 2. Materials and Methods

### 2.1. Human Subjects

Peripheral blood mononuclear cells (PBMCs) were obtained from healthy adults and originate from CTL’s ePBMC^®^ library (CTL, Shaker Heights, OH, USA). Samples were collected at FDA-registered collection centers from IRB-consented healthy human donors by leukapheresis and then were sold to CTL identifying donors by code only while concealing the subjects’ identities. PBMCs were cryopreserved at varying cell densities (10, 5, or 2 × 10^6^ cell per vial) according to previously described protocols [25] and were stored in liquid nitrogen until testing. Details of all human donors included in this manuscript, including demographics and collection dates, are provided in Table S6.

### 2.2. Polyclonal B Cell Stimulation

Detailed methods of thawing, washing, and counting of PBMCs have been previously described [25]. Cells were seeded into polyclonal B cell stimulation cultures within 2 h of thawing. Freshly thawed PBMC samples were resuspended in complete medium consisting of RPMI 1640 (Alkali Scientific, Fort Lauderdale, FL, USA) supplemented with 10% fetal bovine serum (Gemini Bioproducts, West Sacramento, CA, USA), 100 U/mL penicillin, 100 U/mL streptomycin, 2 mM L-glutamine, 1mM sodium pyruvate, 8 mM HEPES (all from Life Technologies, Grand Island, NY, USA), and 50 µM β-mercaptoethanol (Sigma-Aldrich, St. Louis, MO, USA). PBMCs were then stimulated with Human B-Poly-S (CTL) containing TLR7/8 agonist R848 and recombinant human IL-2 [26] at 1–2 × 10^6^ cells/mL in 24-well suspension plates (Corning, Sigma-Aldrich) or 25 cm^2^ or 75 cm^2^ tissue culture flasks and incubated at 37 °C in 5% CO_2_ for five days to promote terminal differentiation of resting B_mem_ into antibody-secreting cells (ASCs) prior to evaluation in ImmunoSpot^®^ assays.

### 2.3. Recombinant Proteins

Recombinant Epstein–Barr virus nuclear antigen 1 (EBNA1) protein was purchased from Serion (Würzburg, Germany). The human cytomegalovirus (HCMV) gH pentamer complex, consisting of gH, gL, UL128, IL130, and UL131A proteins, was purchased from The Native Antigen Company (Kidlington, UK). Recombinant hemagglutinin (rHA) proteins encoding A/California/04/2009 (CA/09, H1N1), A/Texas/50/2012 (TX/12, H3N2), and B/Phuket/3073/2013 (Phuket/13, B/Yam) were acquired from the Center for Vaccines and Immunology (CVI) (University of Georgia (UGA), Athens, GA, USA) and have been described previously [27,28]. Recombinant SARS-CoV-2 spike (S-antigen) protein representing the Wuhan-Hu-1 strain was also acquired from the CVI. Recombinant SARS-CoV-2 nucleocapsid (NCAP) protein was purchased from the Wuhu Interferon Biological Products Industry Research Institute (Wuhu, Anhui Province, China). Importantly, all recombinant proteins used in this study possessed a genetically encoded His affinity tag.

### 2.4. B Cell ImmunoSpot^®^ Assays

The general principle of B cell ELISPOT/FluoroSpot testing is outlined in Supplemental Figure S1. Collectively we refer to both tests as ImmunoSpot^®^, as they are identical except for the last step, the visualization of secretory footprints via enzymatic or fluorescence detection. Specifically, following polyclonal stimulation, as described above, PBMCs were harvested from tissue culture and washed with PBS prior to counting using CTL’s Live/Dead Cell Counting Suite on an ImmunoSpot^®^ S6 Ultimate Analyzer. Cell pellets were resuspended at 3 × 10^6^ live cells per mL (when measuring antigen-specific IgG^+^ ASCs) or 3 × 10^5^ live cells per mL (for detection of pan-Ig-secreting cells), unless otherwise specified in the figure legend, in complete medium and seeded into B cell ImmunoSpot^®^ assays.

For enumeration of Ig-secreting cells irrespective of their antigen specificity (i.e., pan-IgG^+^ ASCs), the cell suspensions were serially diluted 2-fold in duplicates, starting at 3 × 10^4^ live cells per well unless otherwise specified in the figure legend, in round-bottom 96-well tissue culture plates (Corning, Sigma-Aldrich). Subsequently, cells were transferred into ImmunoSpot^®^ assay plates that were pre-coated with anti-κ/λ capture antibody reagents (from CTL) and incubated for 16 h at 37 °C, 5% CO_2_ unless otherwise specified in the figure legend. Plate-bound immunoglobulin (Ig) spot-forming units (SFUs) were subsequently visualized using human-Ig-detecting ImmunoSpot^®^ kits (from CTL) according to the manufacturer’s instructions.

For enumeration of antigen-specific IgG^+^ ASCs, ImmunoSpot^®^ assays were performed using affinity capture coating (ACC) as previously described [29]. Briefly, assay plates were first pre-conditioned with 70% (*v*/*v*) EtOH followed by two washing steps with PBS. Next, wells were coated with purified anti-His tag antibody (from CTL) at 10 µg/mL in Diluent A overnight at 4 °C. The following day, assay plates were washed once with PBS and then coated overnight with His-tag labeled recombinant protein at 10 µg/mL in PBS. Additionally, anti-His-tag-antibody-conditioned wells were coated with 6xHis peptide (GenScript, NJ, USA) at 2 µg/mL as a specificity control for chance recognition by IgG^+^ ASCs. As reported previously [29], modification of the standardized antigen coating procedure was necessary for the gH pentamer complex and was achieved by coating both the anti-His tag antibody and the His-tag-labeled gH pentamer complex at 20 µg/mL in PBS. After overnight incubation of the His-tagged recombinant protein coating solutions at 4 °C, plates were washed once with PBS and then blocked with complete medium for 1 h at room temperature prior to addition of polyclonally stimulated PBMCs at the specified cell numbers per well. Plates were incubated at 37 °C, 5% CO_2_ for 16 h (unless indicated otherwise in the corresponding figure legend) and SFUs were subsequently visualized using human-IgG-detecting ImmunoSpot^®^ kits (from CTL) according to the manufacturer’s instructions.

### 2.5. ImmunoSpot^®^ Image Acquisition and SFU Counting

**Figure 1:**
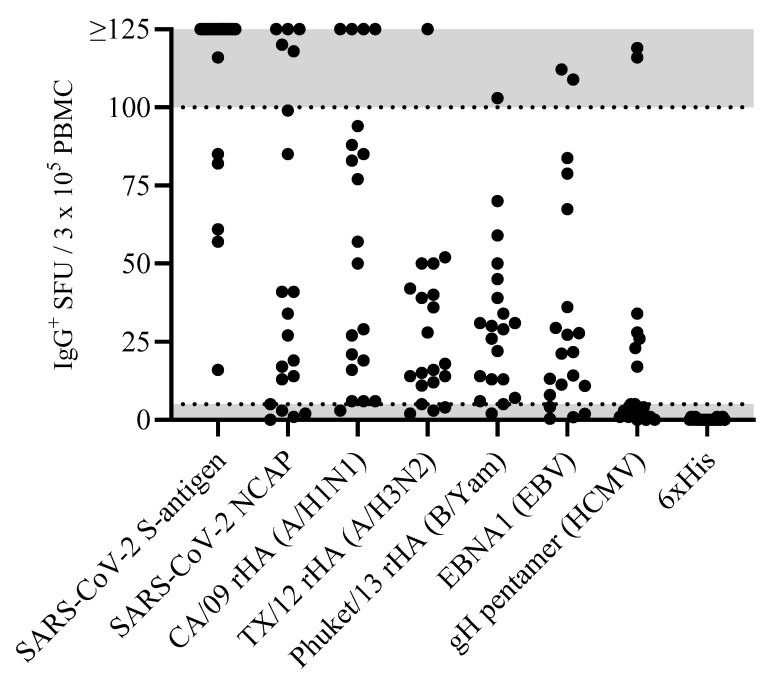
Antigen-specific B_mem_ occur over a wide frequency range in blood. PBMCs from healthy human subjects (n = 20)—each represented by a dot—were tested in ImmunoSpot^®^ assays following in vitro polyclonal stimulation to establish the frequency of B_mem_-derived IgG^+^ ASCs specific for a panel of recombinantly expressed viral antigens (refer to Section 2.3). The data shown represent results obtained when testing PBMCs at single fixed input of 3 × 10^5^ PBMCs per well. Since quantification of wells with >125 antigen-specific spot-forming units (SFUs) is generally imprecise, we defined this as the upper limit for accurate counts. All wells yielding an SFU count exceeding this designated upper limit of quantification were assigned a value of >125 SFUs. Moreover, depending on the distribution and morphology of antigen-specific SFUs in the assay well, we further defined an upper limit of accurate counts at 100 SFUs; denoted by the gray shading in the figure. Similarly, the lower gray shaded region denotes low-frequency responses in which Poisson noise hinders accurate frequency determinations.

Plates were air-dried prior to scanning on an ImmunoSpot^®^ Ultimate S6 Analyzer using either the Fluoro-X suite of ImmunoSpot^®^ software (Version 7.0.28) for FluoroSpot or ImmunoSpot^®^ Studio.SC (Version 1.7.30) for ELISPOT. SFUs were then enumerated using ImmunoSpot^®^ Studio.SC software and B cell IntelliCount^TM^ algorithms [30]. Individual well images were quality controlled as needed. To account for the variable frequencies of pan or antigen-specific ASCs amongst the donors, when applicable, two-fold serial dilutions of the donor PBMCs were evaluated in ImmunoSpot^®^ assays in one or more replicate wells. Only SFU counts within the linear range of the ImmunoSpot^®^ assay were considered for frequency calculations and were subsequently used to extrapolate SFU counts to a designated fixed input (specified in the corresponding figure legend). Relatedly, while the ImmunoSpot^®^ Studio.SC software automatically performs frequency calculations using SFU counts within the user-defined upper and lower bounds, such frequency calculations can also be performed manually. When PBMCs were plated at a fixed input of 3 × 10^5^ PBMCs per well (refer to Figure 1) and the resulting SFU count exceeded the “upper bound” of accurate enumeration, the SFU count was designated as >125. As ImmunoSpot^®^ B cell kits, analyzers, and software proprietary to CTL were used in this study, we refer to this collective methodology as ImmunoSpot^®^.

### 2.6. Statistical Methods

Simple linear regression analysis using GraphPad Prism 10 Version 10.4.0 (San Diego, CA, USA) was performed to calculate R^2^ values and generate the corresponding trend lines. An analysis of variance (ANOVA) with Bonferroni correction for multiple comparisons was performed to compare the frequencies of S-antigen-reactive IgG^+^ ASCs determined using SFU counts originating from the individual serial dilutions performed in singlet or the mean SFU count at each cell input tested in quadruplicate. The mean, standard deviation (SD), and coefficient of variation (expressed as a percentage) between S-antigen-reactive IgG^+^ ASC frequency assessments were calculated using Excel (Microsoft Office 365).

## 3. Results and Discussion

### 3.1. The Need for Testing PBMCs in B Cell ImmunoSpot^®^ Assays in Serial Dilution

Having pioneered an affinity capture coating (ACC) strategy for ImmunoSpot^®^ [29] (illustrated in Supplemental Figure S1A), that facilitates development of B cell ImmunoSpot^®^ assays detecting B_mem_ specific for essentially any antigen, we established such assays for infectious disease-related antigens to which most humans have likely been exposed and thus against which B_mem_ would have been generated. These were the spike (S-antigen) and nucleocapsid (NCAP) proteins of the ancestral Wuhan-Hu-1 strain of SARS-CoV-2, hemagglutinin (HA) proteins representing seasonal influenza A viruses (CA/09, H1N1 or TX/12, H3N2), seasonal influenza B virus (Phuket/13, Yamagata lineage), Epstein–Barr virus (EBNA1), and human cytomegalovirus (gH pentamer complex). Enabled by the new approach, we set out to screen PBMC samples obtained from healthy adult donors for the presence of B_mem_ with reactivity against this antigen panel. Of note, the PBMC samples were collected in May 2022 or later, at which point the majority of donors had likely either been infected by the SARS-CoV-2 virus and/or received one or more doses of a COVID-19 vaccine.

As commonly performed for functional assays, including the traditional (T and) B cell ImmunoSpot^®^ approach, in our initial screening we tested for IgG^+^-antibody-secreting cell (ASC) reactivity to each antigen at a fixed cell number of 3 × 10^5^ PBMCs per well. At this seeding density the PBMC sediment formed a monolayer on the bottom of the 96-well plate, which is ideal for the detection of secretory footprints originating from rare individual lymphocytes using the ImmunoSpot^®^ approach [31]. Moreover, samples were tested in duplicate to counter well-to-well variations that can be expected, particularly when detecting low-frequency events [20,32].

Accounting for the possibility that the frequency of B_mem_ specific for any given antigen in a PBMC test sample can be quite low, it seemed justifiable to test the PBMCs at a cell input close to the monolayer maximum to ensure that even antigen-reactive B_mem_ occurring at low frequencies could be detected. Raw data obtained for 20 representative donors against the eight antigens in our panel are shown in Figure 1. Notably, the number of antigen-reactive IgG^+^ ASCs varied considerably between individual donors, ranging from above the upper limit of quantification (ULOQ) (>125 SFUs per well, see below) to below the lower limit of quantification (LLOQ, <5 SFUs per well). The data also revealed that no donor exhibited a consistently high or low responder status against all antigens in the panel; to the contrary, in line with the premise that each individual possesses a unique exposure history to environmental pathogens [33], the magnitude of viral-antigen-reactive IgG^+^ ASCs detected within and among different donors varied greatly (Supplemental Table S1). Moreover, a spectrum of secretory footprint morphologies was often observed within the same assay well, as well as distinct morphologies in wells coated with different viral antigens (Supplemental Figure S2). Therefore, these data serve to illustrate that testing PBMCs at a fixed number is not suited for reliably establishing the frequency of antigen-reactive IgG^+^ B_mem_ cells in ImmunoSpot^®^ assays.

**Figure 2:**
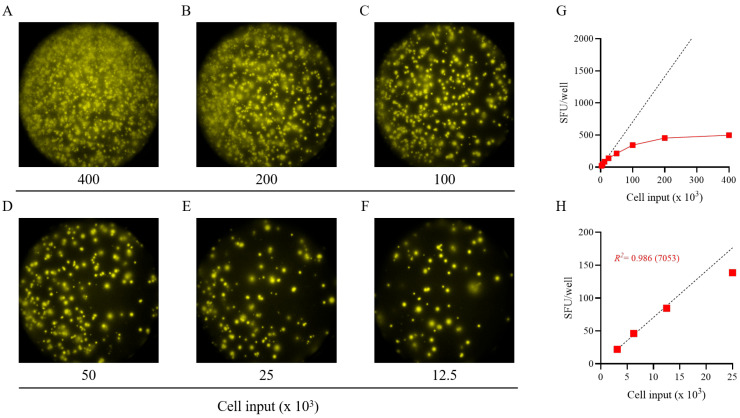
Establishing the frequency of S-antigen-specific B_mem_-derived IgG^+^ ASCs using a serial dilution approach. PBMCs from a healthy human subject, collected in the post-COVID era, were tested in an ImmunoSpot^®^ assay using a two-fold serial dilution approach to determine the frequency of SARS-CoV-2 S-antigen-specific IgG^+^ ASCs following 5 days of in vitro polyclonal stimulation. (**A**–**F**) Representative well images are shown for each of the specified cell inputs. Note: crowding of spot-forming units (SFUs) and elevated background membrane staining resulting from an ELISA effect were present in wells input with >1 × 10^5^ PBMCs. (**G**) SFU counts per well (red symbols) vs. cell input over the entire cell dilution range tested. Deviation from linearity was evident at values >100 SFUs per well, denoted by the dashed line, representing the theoretical linear relationship between SFU counts and PBMCs plated. (**H**) Linearity between SFU counts and cell input in wells seeded with <2.5 × 10^4^ PBMCs; R^2^ value denotes the goodness of fit of the data. Notably, for this particular donor and antigen combination the mean SFU count from the 2.5 × 10^4^ PBMC input underestimated the frequency of S-antigen-specific IgG^+^ ASCs, denoted in parentheses as the number of S-antigen-specific IgG^+^ ASCs per 10^6^ PBMCs. Note: refer to Supplemental Figure S4 in which the linearity between cell input and SFU counts is shown for an additional 11 test subjects.

To more precisely determine the frequency of antigen-reactive IgG^+^ B_mem_ in donors that initially yielded a result above the assay’s ULOQ, we re-evaluated such antigen/PBMC combinations using a 2-fold serial dilution approach. As shown for a representative sample in Figure 2, a close to perfect linear relationship was seen between the number of SARS-CoV-2 spike (S-antigen)-reactive IgG^+^ secretory footprints (spot-forming units, SFUs) counted and PBMCs plated, but only when the antigen-reactive SFU counts were less than 100 per well (Figure 2H); at higher cell inputs for this antigen/donor combination the confluence of SFUs and elevated membrane staining (occurring due to an ELISA effect in which antibodies escape into the supernatant and then subsequently bind to the antigen-coated membrane distally from the source ASCs—visible in Figure 2A–C) resulted in undercounting of individual SFUs (Figure 2G). The data show that secretory footprints of individual ASCs could be accurately detected under 100 SFUs per well, constituting the ULOQ for this particular donor. For other donors, owing either to variable SFU morphologies and/or fortuitously finding the optimal serial dilution window, the ULOQ can be slightly lower or higher than 100 SFUs per well. Taking this into consideration, we denoted a “gray zone” as the ULOQ in Figure 1. Nevertheless, for most antigen-specific assays (including S-antigen), the linearity region of the curve rarely exceeds 125 SFUs per well. 

The LLOQ can be conservatively set at 5 SFUs per well. According to Poisson’s rule, which applies when measuring antigen-specific lymphocytes [20], the well-to-well variation in SFUs generated by rare ASCs increases dramatically at lower precursor frequencies. Therefore, in such instances, a multitude of replicates would be required to firmly establish their actual abundance in a test sample. Importantly, only in a narrow window, between 10 and 100 SFUs per well, are antigen-specific SFU counts inherently reliable for frequency calculations. Depending on the frequency of antigen-specific ASCs in a test sample, as well as the resulting secretory footprint morphologies, the ideal range of SFUs for frequency calculations will be unique to each PBMC/antigen combination and hence can only be consistently established using a serial dilution testing approach. Supplemental Figure S3 illustrates both the high degree of variability in S-antigen-reactive B cells among 12 donors and that a serial dilution approach permits the establishment of a linear range for cell input vs. SFU counts from which the frequencies can be calculated. Supplemental Figure S4 depicts the linear range for 11 of these 12 donors, with regression lines and frequency extrapolations shown. Supplemental Table S2 further shows the reproducibility of S-antigen-reactive IgG^+^ ASC frequencies—even when they varied considerably between the test samples. While these data are for S-antigen, in the interim we have been implementing serial-dilution-based frequency assessments for most of the antigens shown in Figure 1, confirming the generalizability of the finding for healthy donors. However, whether this is also applicable when testing samples obtained from elderly and immune-compromised subjects remains an open question.

**Figure 3:**
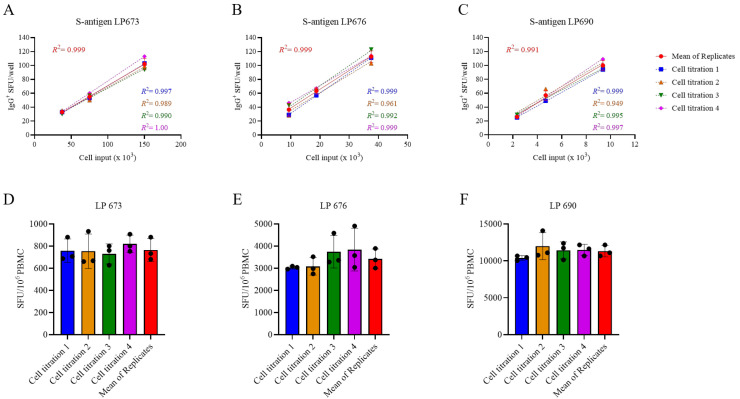
Measurements of S-antigen-specific IgG^+^ ASC frequencies using a singlet serial dilution approach are similar to those based on the mean of quadruplicates. PBMCs from three healthy human subjects, collected in the post-COVID era, were evaluated for S-antigen-specific IgG^+^ ASCs following 5 days of in vitro polyclonal stimulation using a serial dilution approach and four replicate wells at each cell input. (**A**–**C**) S-antigen-specific IgG^+^ SFU counts occurring within the linear range for each donor are plotted. The mean ± SD SFU counts from the four replicate wells, along with the regression line and R^2^ values, are in red. Additionally, datapoints originating from the four singlet serial dilutions, respectively, were used for linear regression analysis and the corresponding trend lines and R^2^ values are denoted in the corresponding colors indicated in the figure inset. (**D**–**F**) The frequency of S-antigen-specific IgG^+^ ASCs was then calculated using three consecutive datapoints from a singlet serial dilution or the mean SFU count at the corresponding cell inputs and then extrapolated to 10^6^ PBMCs. Importantly, no statistically significant differences were detected between the extrapolated datapoints originating from the individual singlet serial dilutions or the mean of quadruplicate wells using an analysis of variance (ANOVA) with Bonferroni correction for multiple comparisons.

Serial dilutions are therefore necessary for achieving accurate B_mem_ frequency measurements in PBMC samples in which antigen-reactive ASCs are abundant, but this increases the number of PBMCs required for testing. Observing the close to perfect linear relationship between PBMC numbers plated and SFUs detected per well, we sought to test the hypothesis that the serial dilution strategy itself could substitute for the reliance on technical replicates. In Figure 3A–C, PBMCs from three SARS-CoV-2 S-antigen-reactive subjects were serially diluted in four replicate wells for each dilution—SFU counts are shown for the linear range of 10 to 100 SFUs per well only. The mean SFU counts at the designated cell inputs were first calculated, and it was established that the mean SFU counts originating from 2-fold serially diluted PBMCs closely followed a linear function. Moreover, very similar regression lines were also obtained when the same calculations were performed using each of the replicates separately. Moreover, frequencies were calculated by extrapolation using either the individual SFU counts within a singlet serial dilution or using the mean counts from the quadruplicates (Figure 3D–F). In each instance, the frequency of S-antigen-reactive IgG^+^ ASCs per 10^6^ PBMCs was not significantly different between the respective singlet serial dilutions nor were they different from the frequency determined using the mean SFU count from the four replicate wells at each cell dilution. The conclusion that singlet serial dilutions enable accurate frequency determinations was also confirmed for several additional antigens and for determining the frequency of ASCs producing different classes of immunoglobulin (IgA, IgM, or IgG) (Supplemental Figure S5). Collectively, these data demonstrate that serial dilutions performed in single wells yield nearly equivalent data compared to results obtained from serial dilutions performed in quadruplicate; however, the former approach requires only a quarter as many PBMCs, representing the first substantial saving on blood volumes needed for routine testing.

### 3.2. Multiplexing B Cell ImmunoSpot^®^ Assays

Immunoglobulins (Igs) occur in four major classes (IgA, IgE, IgG, and IgM) and there are four IgG subclasses (IgG1, IgG2, IgG3, and IgG4). While all of these molecules share the task of specific antigen recognition, they can fundamentally differ in the effector functions they elicit upon binding to antigen. Comprehensive immune monitoring therefore needs to account for each of these Ig classes and subclasses, including the B_mem_ that are pre-committed to secrete them. Traditional ELISPOT assays measure one analyte at a time: one would need to run four single-color assays to cover all the Ig classes, plus an additional four single-color assays to segregate each of the IgG subclasses, necessitating increased PBMC utilization. Multiplexing the detection of Ig classes and/or IgG subclasses is the most practical solution to this requirement and—as we will show in the following—can be accomplished without increasing the need for additional PBMC test material.

**Figure 4:**
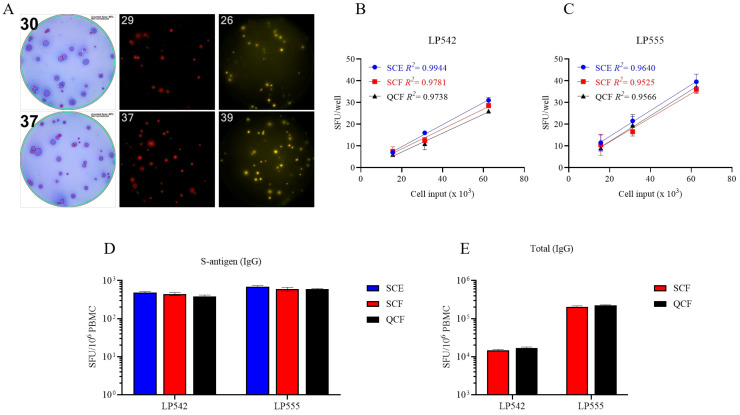
Single-color ELISPOT and multicolor FluoroSpot assays have similar sensitivities for detecting S-antigen-specific B_mem_-derived IgG^+^ ASCs. (**A**–**C**) PBMCs from two convalescent donors with PCR-verified SARS-CoV-2 infection were polyclonally stimulated and S-antigen-specific B_mem_-derived IgG^+^ ASCs detected in single-color ELISPOT (SCE), single-color FluoroSpot (SCF), or four (quad)-color (QCF) FluoroSpot assays. (**A**) Representative well images from S-antigen-specific ELISPOT or FluoroSpot assays. Cell input was ~6.25 × 10^4^ PBMCs for both donors. (**B**,**C**) S-antigen-specific IgG^+^ SFU counts occurring within the linear range for both ELISPOT and FluoroSpot assays are plotted for each donor. The mean ± SD of replicate wells, along with the regression line and R^2^ values, are denoted in the corresponding colors indicated in the figure insets. (**D**,**E**) SFU counts occurring within the linear range of ELISPOT and/or FluoroSpot assays were used to calculate the frequency of S-antigen-specific or pan (total) IgG^+^ ASCs and are expressed per 10^6^ PBMCs. Note, the cell incubation period for the depicted ELISPOT and FluoroSpot assays was shortened to 5 h to reduce background membrane staining that often occurs within ELISPOT assays when frequencies of antigen-reactive ASCs are elevated.

While four-color B cell ImmunoSpot^®^ analysis is already commercially available [19], so far its sensitivity for detecting the individual antigen-specific Ig analytes has not been formally established versus their single-color measurements. Multiplexing in FluoroSpot analysis is currently limited to four analytes owing to the requirement that each fluorescence-tagged detection antibody possess distinct excitation/emission properties that do not overlap, so that each can be visualized without leaking into the other’s detection channels and therefore permitting automated single-color counting in each color plane without the need for compensation [19]. In systematic studies we have established here that traditional single-color enzymatic ELISPOT assays and four-color fluorescent analysis provide indistinguishable antigen-specific IgG SFU counts within the linear range (Figure 4); however, in ELISPOT the linearity in S-antigen-specific SFU counts broke down earlier than FluoroSpot in large part due to the increased size of individual secretory footprints and elevated membrane staining in wells with a high density of SFUs—both attributable to enzymatic amplification. Similarly, we previously demonstrated that pan (total) Ig SFU counts were also equivalent between single-color enzymatic ELISPOT and single- or four-color FluoroSpot assays [25].

With this notion in mind, we systematically studied PBMCs obtained from healthy human donors in order to determine the frequency of ASCs that secrete the four Ig classes and the four IgG subclasses. Following five days of in vitro polyclonal stimulation, the donors’ PBMCs were plated at 2 × 10^5^ cells per well and then serially diluted 2-fold. The results shown in Supplemental Table S3 established that (a) even for a single Ig class or IgG subclass, the frequency of pan-Ig ASCs can vary substantially between healthy individuals, and (b) that in all individuals IgM-, IgG- and IgG1-secreting ASCs substantially outnumbered ASCs secreting other classes/subclasses. In most instances the SFU counts originating from IgM^+^, IgG^+^, or IgG1^+^ ASCs were above the linear range for accurate frequency calculations at the same PBMC inputs at which ASCs secreting the rarer classes/subclasses (IgA, IgG2, IgG3, and IgG4) were exceedingly infrequent, if not undetectable. Lastly, these studies established that—except if one is particularly interested in the rarer Ig classes and subclasses—starting the serial dilution at 2–3 × 10^4^ PBMCs per well is sufficient for multiplexed pan-Ig measurements.

Systematic four-color ImmunoSpot^®^ studies conducted to establish the frequency of B_mem_-derived ASCs with reactivity for a SARS-CoV-2 S-antigen representing the prototype Wuhan-Hu-1 strain were consistent with results shown in Figure 1 (Yao et al., manuscript in preparation). Collectively, these data confirmed the prevalence of the IgG class, and specifically the IgG1 subclass, within the antigen-specific ASC compartment.

Based on the data presented, and all the experience we have gained so far, we recommend, for initial B_mem_ characterization, to perform tests in which the PBMCs are serially diluted starting at 1–3 × 10^5^ cells per well for each antigen and at 1–3 × 10^4^ PBMCs per well for the pan-Ig assay. Since these tests can each be conducted using a single well serial dilution approach without compromising the resolution of the results, and because the detection of four Ig classes or IgG subclasses can be multiplexed using four-color detection systems, even when starting with a 3 × 10^5^ PBMC input, 1.2 × 10^6^ cells from the polyclonal stimulation cultures are sufficient for measuring all four Ig classes and four IgG subclasses per antigen with an additional 1.2 × 10^5^ cells needed for establishing the frequency of ASCs producing each of the four Ig classes or IgG subclasses (irrespective of antigen specificity). The recommended plate layout for routine PBMC testing in B cell ImmunoSpot^®^ assays (Supplemental Figure S6) accommodates frequency determinations for both antigen-specific and pan-Ig B_mem_ of all classes and subclasses despite the considerable variability of frequencies in which such B_mem_ occur in blood, except for those being present at very low frequencies.

### 3.3. Extending the Lower Limit of Detection

As can be seen in Figure 1, when tested at 3 × 10^5^ PBMCs per well, there are still some donors in whom antigen-specific IgG^+^ ASCs were not detected above the positivity threshold (>5 SFUs per 3 × 10^5^ PBMCs). Such an outcome may reflect two fundamentally different scenarios: either such donors have not been exposed to the antigen (true negative) or the frequency of B_mem_ reactivity for the antigen in question was below the detection limit of the assay as performed for this particular donor (false negative). Owing to the heterogeneous and often unverifiable antigen exposure histories of human subjects, determining the distinction between a true negative vs. a false negative test result is not a simple task. SARS-CoV-2 S-antigen and NCAP provide here a rare opportunity to distinguish between immunologically naïve and antigen-exposed individuals. As shown in Supplemental Table S4, subjects whose PBMCs were cryopreserved before the introduction of the SARS-CoV-2 virus into the human population exhibited negligible IgG^+^ ASC reactivity when 1 × 10^5^ or 2 × 10^5^ PBMCs were tested against SARS-CoV-2 S-antigen or NCAP, respectively. Such samples serve as true negatives and yielded the expected results. In contrast, donors who had either recovered from verified SARS-CoV-2 infection or completed the primary COVID-19 mRNA vaccination series possessed clearly elevated frequencies of S-antigen reactive B_mem_-derived IgG^+^ ASCs and provided 100% diagnostic specificity when tested at 1 × 10^5^ PBMCs or lower inputs per well. However, these positive test results were seen using samples isolated within half a year following verified infection or vaccination and raised the question of whether testing 2 × 10^5^ PBMCs would suffice for reliably detecting S-antigen or NCAP-reactive B_mem_ in samples from subjects whose prior infection and/or vaccination history was unknown.

With this specific question in mind, cryopreserved PBMCs (collected between May and October 2022) were subjected to polyclonal stimulation and evaluated for B_mem_-derived IgG^+^ ASC reactivity against SARS-CoV-2 S-antigen and NCAP representing the ancestral Wuhan-Hu-1 strain (Supplemental Table S5). Owing to the elevated frequency of S-antigen-reactive B_mem_-derived IgG^+^ ASCs detected in earlier experiments (Figure 1), these samples were exclusively tested using a singlet serial dilution approach starting at 2 × 10^5^ PBMCs per well. However, to account for the possibility that NCAP-reactive B_mem_ frequencies could be low, we tested these samples using either a singlet serial dilution approach starting at 2 × 10^5^ or by seeding replicate wells with 5 × 10^5^ PBMCs. Notably, B_mem_-derived IgG^+^ ASC reactivity against the S-antigen was readily apparent in 100% of these donors (n = 10) and supports the notion that S-antigen-reactive B_mem_ were generated as a consequence of prior infection(s) and/or vaccination(s). Half (5/10) of these donors, however, did not exhibit clearly elevated numbers of NCAP-reactive B_mem_-derived IgG^+^ ASCs when tested at an initial starting input of 2 × 10^5^ PBMCs per well. Had these five donors indeed avoided prior SARS-CoV-2 virus infection despite its wide spread early in 2022 [34], or were the frequencies of NCAP-reactive B_mem_ in such PBMC samples merely below the detection limit of the assay as performed? Only through modifying the testing procedure and seeding 5 × 10^5^ PBMCs per well to augment the assay’s detection limit could this question be addressed. Among the donors that yielded <5 NCAP-reactive IgG^+^ SFUs in wells seeded with 2 × 10^5^ PBMCs, four of five donors demonstrated an increased number of NCAP-reactive IgG^+^ SFUs in wells seeded with 5 × 10^5^ PBMCs. Nevertheless, the resulting SFU counts were not necessarily increased by 2.5-fold as might be expected. However, when the SFU counts from the four replicate NCAP-coated wells seeded with 5 × 10^5^ PBMCs were aggregated together, entailing a 10-fold increase in the number of PBMCs tested in the assay, it became more evident that 8 of 10 donors had likely been previously infected; however, this cannot be definitively confirmed. Furthermore, when testing PBMCs at high cell inputs, and particularly when aiming to detect very low frequencies of antigen-specific ASCs, it is advisable to incorporate an irrelevant antigen into the test to serve as a comparator for “chance” reactivity.

As such, B cell ImmunoSpot^®^ assays are capable of detecting individual ASCs so long as (a) these cells do not compete for real estate on the membrane on which their respective secretory footprints are being captured (as shown above, this is a limitation readily overcome by performing serial dilutions when measuring high-frequency ASC populations) and (b) the PBMCs do not pile up in layers on the membrane. In the latter case of cell crowding, bystander cells in the lower stratum can be expected to hinder the generation of secretory footprints on the antigen-coated membrane by ASCs in the upper strata. From direct visualization of PBMCs on the membrane, we already know that cell inputs of >1 × 10^6^ PBMCs per well exceed a single stratum (i.e., a monolayer) when input into a standard 96-well assay plate [31]. To experimentally address how many PBMCs can be plated per well before cell crowding starts to interfere with detection of ASCs at single cell resolution, we admixed polyclonally simulated PBMCs (that contained ASCs at an optimal “Goldilocks number”, i.e., ~50 SFUs per well) with increasing numbers of autologous unstimulated PBMCs (that do not contain IgG^+^ ASCs). The results are shown in Supplemental Figure S7, indicating that PBMC inputs exceeding 5 × 10^5^ per well can undermine the ability to discern individual secretory footprints. Therefore, if extending the lower limit of detection is intended, the number of PBMCs interrogated can be indefinitely increased by testing additional replicates, each at 5 × 10^5^ PBMCs per well.

Collectively, the data presented so far suggest that testing of uncharacterized PBMCs for antigen-specific ASCs should start at 3–5 × 10^5^ PBMCs per well and progress in a 2-fold serial dilution. This approach should permit reliable detection and accurate quantification of ASC populations existing at intermediate to high frequencies. If a donor is in the low/ambiguous frequency range, subsequent re-testing at 5 × 10^5^ PBMCs per well in replicates will enable extending the limit of detection and of quantification. Such a strategy requires, however, that additional vials of cryopreserved PBMCs are set aside (see below). As an alternative “fail-safe” approach, to accommodate the scenario in which B_mem_ frequencies in the study cohort could also exist at low frequencies, in the first test one could perform a serial dilution in singlet wells and also set up replicate wells with an input of 5 × 10^5^ PBMCs. In such a testing approach, the serial dilution would adequately cover the intermediate to high ASC frequency range, while the replicate wells seeded with the highest cell input would enable low-frequency measurements. We confirmed the utility of such a “fail-safe” approach for detecting low frequencies of S-antigen-reactive IgA^+^ or IgG4^+^ ASCs in a cohort of COVID-19-mRNA-vaccinated donors, whereas the frequency of the more abundant S-antigen-reactive IgG1^+^ ASCs was determined using a serial dilution approach and a lower starting cell input of 2 × 10^5^ PBMCs.

### 3.4. Targeted Cryopreservation of PBMCs for Testing (and Re-Testing) in B Cell ImmunoSpot^®^ Assays

In previous work we have established protocols according to which PBMCs can be cryopreserved without loss of functionality for later testing in B cell ImmunoSpot^®^ assays [19,25] and we have shown the high reproducibility of the data (CV < 20%) when different aliquots of the same sample are tested on different days, even by different investigators [20]. Such robust assay performance is essential for the ability to (a) test clinical samples in central laboratories far from sites where they were collected, (b) test samples independent of the time point of collection, (c) test PBMCs of different donors side-by-side in a single high-throughput experiment avoiding inter-assay variations of results, (d) reproduce results by repeatedly testing aliquots of the same blood draw, and (e) extend the results of the first screening run if needed or intended, e.g., to lower detection limits, or to perform subsequent B cell cross-reactivity or affinity spectrum analysis by ImmunoSpot^®^ [35,36]. For the latter two options it is essential to first establish the “Goldilocks number” that yields ~50 SFUs per well for a given antigen/sample combination. Cryopreservation is also essential (f) for generating reference PBMCs with established B_mem_ frequencies for antigens of interest [12], as well as for (g) the ability to validate B cell ImmunoSpot^®^ assays [20].

Cryopreservation of PBMCs for testing in functional assays is already well established and is traditionally performed at 10 million PBMCs per vial. The data shown above, however, suggest that only 1–2 × 10^6^ PBMCs following the 5-day polyclonal stimulation would be sufficient to establish the frequency of B_mem_ with reactivity for an antigen, even when the assessment extends to all four Ig classes and four IgG subclasses. Even if the PBMCs would need to be re-tested, e.g., to extend the lower detection limit, only a few million more cells at most (far less than 10 million) would be needed. The question therefore arose as to whether PBMCs can be cryopreserved in lower numbers per cryovial than 10 million to avoid the waste of precious cell material.

To address this question, PBMCs collected by leukapheresis were either directly subjected to polyclonal stimulation immediately upon receipt, without cryopreservation, i.e., were tested “fresh”, or following cryopreservation in aliquots containing 10, 5, or 2 × 10^6^ PBMCs per vial. To allow for batch testing of multiple donors’ samples in parallel, cryopreserved aliquots were thawed two weeks or more after being generated, subjected to polyclonal stimulation, and then tested for ASC activity in ImmunoSpot^®^ assays. Pan-Ig and antigen-specific ASC frequencies were established using the singlet two-fold serial dilution strategy described above. In Table 1, the frequencies of S-antigen-reactive IgG^+^ ASCs are represented as the percentage of all IgG^+^ ASCs. The results are essentially identical for fresh and frozen cells and, notably, irrespective of the number of PBMCs cryopreserved per vial. Collectively, these data indicate, at least for healthy adult donors, that cryopreserving a custom number of PBMCs is feasible. However, whether this conclusion can be extended to donors with different disease states or those undergoing various therapies would need to be verified in each specific clinical scenario.

The above experiment also provided insights into cell losses associated with the cryopreservation of PBMCs obtained from healthy adult donors at various cell densities. As seen in Table 2, the viabilities of PBMCs frozen at the various cell densities upon thawing and following the polyclonal stimulation were indistinguishable and mirrored what was observed using freshly isolated cell material. Data accumulated over the years documenting cell recovery following the in vitro polyclonal stimulation procedure when starting with samples cryopreserved at 10 million PBMCs per vial confirm the conclusion that with rare exceptions at least 50% of the PBMCs can be recovered after cryopreservation followed by a 5-day polyclonal stimulation culture (Supplemental Figure S8B). This finding may serve as a guide for planning B cell ImmunoSpot^®^ assays cryopreserving custom numbers of PBMCs for optimized cell utilization.

We have established here that PBMCs can be cryopreserved short-term without impaired B cell function irrespective of cell numbers frozen per vial, but it will be important to learn whether this also applies for long-term storage. While we have not studied this question in due detail yet, we have encouraging data suggesting that samples cryopreserved at 10 million PBMCs per vial fully maintain their B cell functionality in liquid nitrogen for at least 4 years; we have obtained blood by leukapheresis from donors after PCR-verified SARS-CoV-2 infection early on (starting in late 2020) and have repeatedly tested aliquots of them since, reproducing the original results obtained back then even after 4 years (Kirchenbaum, work in progress). Notably, for successful long-term storage of cryopreserved cell material it is important to avoid glass phase transitions [37,38] due to partial warming up of cells when aliquots are handled and/or removed from the liquid nitrogen storage tank.

## 4. Summary and Conclusions

As the amount of blood/PBMCs available is one of the primary limitations for conducting cell-based immune monitoring, we focused in this publication on the logistics of how to optimize PBMC utilization for detection of antigen-specific B_mem_ cells using ImmunoSpot^®^ assays. We have established that, while testing PBMCs in serial dilution is necessary for defining the frequency of antigen-specific ASCs when they are abundant, doing so in singlet wells provides in most cases results that are just as accurate as tests performed with additional technical replicates. We have also established that four-color multiplexed Ig class or subclass determinations are an additional means for maximizing PBMC utilization without compromising on assay sensitivity. When testing healthy donors for B_mem_ specific for antigens representing ubiquitous and commonly encountered viruses, we conclude that an initial screening approach that starts at 3–5 × 10^5^ PBMCs per well, with subsequent serial dilutions, will accommodate the majority of cases where B_mem_ frequencies are in the intermediate to high range. This can readily be accomplished using 0.6–1 × 10^6^ PBMCs per donor following the 5-day polyclonal stimulation protocol, which is obtainable from 2 mL, or more conservatively 3 mL, of blood even following cryopreservation. If, for some donors, B_mem_ frequencies are below the assay’s lower limit of quantification (LLOQ) when tested using the proposed approach (i.e., <10 SFUs per 3–5 × 10^5^ PBMCs), yet it is desired to augment the LLOQ, thawing additional aliquots and re-testing the sample(s) at 5 × 10^5^ PBMCs per well in replicate wells is a viable strategy. The calculable need for cells, and the predictable recovery of PBMCs following their cryopreservation and subsequent polyclonal stimulation, makes it possible to customize the number of PBMCs cryopreserved per vial and achieve optimal sample utilization for downstream testing. Notably, while all findings reported here were made using PBMCs obtained from healthy adult donors, we hope that our findings will succeed in catalyzing similar studies to be undertaken using samples obtained from pediatric and/or geriatric subjects, including immune-suppressed donors and various diseases as well (in all of which PBMCs are particularly limiting), as such information should be taken into consideration for achieving optimal sample utilization in the context of a clinical trial.

## Figures and Tables

**Table 1:** Frequency of S-antigen-specific IgG^+^ ASCs in fresh or cryopreserved PBMC samples. PBMCs from six healthy human subjects were subjected to 5 days of in vitro polyclonal stimulation either immediately upon receipt, without cryopreservation, or following cryopreservation in aliquots containing 10, 5, or 2 × 10^6^ PBMCs per vial and then evaluated in B cell ImmunoSpot^®^ assays to quantify the number of S-antigen-specific and pan-IgG^+^ ASCs using a singlet two-fold serial dilution approach (refer to Section 2.4). Data are expressed as the frequency of S-antigen-specific ASCs amongst all IgG-secreting cells. The mean, standard deviation (SD), and coefficient of variation (CV) between S-antigen-specific IgG^+^ ASC frequencies in the donors’ PBMC samples cryopreserved at different cell densities are denoted.

		Freezing Density (Cells/mL)					
Donor	Fresh	10 × 10^6^	5 × 10^6^	2 × 10^6^	Mean	SD	CV
LP734	2.07%	2.16%	1.74%	1.90%	1.9%	0.21%	10.8%
LP739	0.93%	1.44%	1.31%	1.50%	1.4%	0.10%	6.8%
LP801	4.51%	5.41%	4.05%	4.67%	4.7%	0.68%	14.5%
LP803	3.15%	3.00%	4.40%	3.86%	3.8%	0.71%	18.9%
LP804	1.01%	1.92%	1.89%	1.91%	1.9%	0.01%	0.8%
LP806	1.07%	1.52%	1.53%	1.18%	1.4%	0.20%	13.9%

**Table 2:** Viability and cell recovery of PBMCs before and after polyclonal stimulation. Viability and cell recovery of freshly isolated or PBMCs cryopreserved at different cell densities (refer to Section 2.1 and Table 1).

Donor	Fresh		Freezing Density (×10^6^)	Frozen		
	Viability Before 5-Day Culture	Viability After 5-Day Culture		Viability Upon Thawing	Viability After 5-Day Culture	Cell Recovery After Thawing
LP734	98%	70%	10	96%	66%	81%
			5	96%	69%	84%
			2	97%	65%	81%
LP739	98%	73%	10	96%	69%	70%
			5	98%	67%	77%
			2	98%	71%	78%
LP801	97%	72%	10	94%	72%	58%
			5	95%	69%	68%
			2	95%	67%	61%
LP803	97%	83%	10	95%	66%	55%
			5	97%	72%	63%
			2	97%	73%	56%
LP804	97%	85%	10	92%	69%	60%
			5	93%	71%	68%
			2	93%	72%	59%
LP806	97%	76%	10	94%	79%	71%
			5	95%	79%	64%
			2	95%	78%	70%

## Data Availability

The data generated in this study will be made available by the authors, without undue reservation, to any qualified researcher.

## Supplementary Materials

The following supporting information can be downloaded at: https://www.mdpi.com/article/10.3390/vaccines13070765/s1. Figure S1. B cell ImmunoSpot^®^ assays for detection of antigen-specific or pan (total) IgG secretory footprints; Figure S2. Bmem-derived IgG+ secretory footprints occur with diverse morphologies in ImmunoSpot^®^ assays detecting ASCs specific for different antigens; Figure S3. Serial dilution permits reliable assessment of variable S-antigen-reactive IgG+ ASC frequencies; Figure S4. Quantification of S-antigen-reactive IgG+ ASCs using a singlet serial dilution approach; Figure S5. Quantification of pan (total) Ig ASCs using a singlet serial dilution approach is similar to that based on the mean of quadruplicates; Figure S6. Plating approach for optimal cell utilization or improving the limit of detection in B cell ImmunoSpot^®^ assays; Figure S7. PBMC inputs exceeding 5 × 10^5^ per well can undermine the ability to discern individual antigen-specific secretory footprints; Figure S8. PBMC viability and cell recovery following thawing and after polyclonal stimulation; Table S1. Viral-antigen-specific Bmem-derived IgG+ ASC frequencies vary considerably between individual donors; Table S2. Day-to-day variability of S-antigen-reactive IgG+ SFU counts; Table S3. Variable frequencies of ASCs producing the different Ig classes and IgG subclasses following polyclonal stimulation of PBMCs; Table S4. SARS-CoV-2 Spike and Nucleocapsid antigen-specific IgG+ ASCs are detectable only in antigen-exposed individuals; Table S5. Lowering the detection limit of SARS-CoV-2 NCAP-specific Bmem-derived IgG+ ASCs; Table S6: donor demographics.

## References

[1] Akkaya M., Kwak K., Pierce S.K. (2020). B cell memory: Building two walls of protection against pathogens. Nat. Rev. Immunol..

[2] Blandino R., Baumgarth N. (2019). Secreted IgM: New tricks for an old molecule. J. Leukoc. Biol..

[3] de Sousa-Pereira P., Woof J.M. (2019). IgA: Structure, Function, and Developability. Antibodies.

[4] Lawrence M.G., Woodfolk J.A., Schuyler A.J., Stillman L.C., Chapman M.D., Platts-Mills T.A. (2017). Half-life of IgE in serum and skin: Consequences for anti-IgE therapy in patients with allergic disease. J. Allergy Clin. Immunol..

[5] Morell A., Terry W.D., Waldmann T.A. (1970). Metabolic properties of IgG subclasses in man. J. Clin. Investig..

[6] Pyzik M., Kozicky L.K., Gandhi A.K., Blumberg R.S. (2023). The therapeutic age of the neonatal Fc receptor. Nat. Rev. Immunol..

[7] Amanna I.J., Carlson N.E., Slifka M.K. (2007). Duration of humoral immunity to common viral and vaccine antigens. N. Engl. J. Med..

[8] Fooksman D.R., Jing Z., Park R. (2024). New insights into the ontogeny, diversity, maturation and survival of long-lived plasma cells. Nat. Rev. Immunol..

[9] Lightman S.M., Utley A., Lee K.P. (2019). Survival of Long-Lived Plasma Cells (LLPC): Piecing Together the Puzzle. Front. Immunol..

[10] Robinson M.J., Ding Z., Dowling M.R., Hill D.L., Webster R.H., McKenzie C., Pitt C., O’Donnell K., Mulder J., Brodie E. (2023). Intrinsically determined turnover underlies broad heterogeneity in plasma-cell lifespan. Immunity.

[11] Seow J., Graham C., Merrick B., Acors S., Pickering S., Steel K.J., Hemmings O., O’Byrne A., Kouphou N., Galao R.P. (2020). Longitudinal observation and decline of neutralizing antibody responses in the three months following SARS-CoV-2 infection in humans. Nat. Microbiol..

[12] Wolf C., Köppert S., Becza N., Kuerten S., Kirchenbaum G.A., Lehmann P.V. (2022). Antibody Levels Poorly Reflect on the Frequency of Memory B Cells Generated following SARS-CoV-2, Seasonal Influenza, or EBV Infection. Cells.

[13] Xiang T., Liang B., Fang Y., Lu S., Li S., Wang H., Li H., Yang X., Shen S., Zhu B. (2021). Declining Levels of Neutralizing Antibodies Against SARS-CoV-2 in Convalescent COVID-19 Patients One Year Post Symptom Onset. Front. Immunol..

[14] Palm A.E., Henry C. (2019). Remembrance of Things Past: Long-Term B Cell Memory After Infection and Vaccination. Front. Immunol..

[15] Inoue T., Moran I., Shinnakasu R., Phan T.G., Kurosaki T. (2018). Generation of memory B cells and their reactivation. Immunol. Rev..

[16] Victora G.D., Nussenzweig M.C. (2022). Germinal Centers. Annu. Rev. Immunol..

[17] Matz H.C., McIntire K.M., Ellebedy A.H. (2023). Persistent germinal center responses: Slow-growing trees bear the best fruits. Curr Opin. Immunol..

[18] Kirchenbaum G.A., Pawelec G., Lehmann P.V. (2025). The Importance of Monitoring Antigen-Specific Memory B Cells, and How ImmunoSpot Assays Are Suitable for This Task. Cells.

[19] Fecher P., Caspell R., Naeem V., Karulin A.Y., Kuerten S., Lehmann P.V. (2018). B Cells and B Cell Blasts Withstand Cryopreservation While Retaining Their Functionality for Producing Antibody. Cells.

[20] Lehmann P.V., Karulin A.Y., Becza N., Yao L., Liu Z., Chepke J., Maul-Pavicic A., Wolf C., Köppert S., Valente A.V. (2025). Theoretical and practical considerations for validating antigen-specific B cell ImmunoSpot assays. J. Immunol. Methods.

[21] Phelps A., Pazos-Castro D., Urselli F., Grydziuszko E., Mann-Delany O., Fang A., Walker T.D., Guruge R.T., Tome-Amat J., Diaz-Perales A. (2024). Production and use of antigen tetramers to study antigen-specific B cells. Nat. Protoc..

[22] Boonyaratanakornkit J., Taylor J.J. (2019). Techniques to Study Antigen-Specific B Cell Responses. Front. Immunol..

[23] Czerkinsky C.C., Nilsson L.Å., Nygren H., Ouchterlony Ö., Tarkowski A. (1983). A solid-phase enzyme-linked immunospot (ELISPOT) assay for enumeration of specific antibody-secreting cells. J. Immunol. Methods.

[24] Sedgwick J.D., Holt P.G. (1983). A solid-phase immunoenzymatic technique for the enumeration of specific antibody-secreting cells. J. Immunol. Methods.

[25] Yao L., Becza N., Maul-Pavicic A., Chepke J., Kirchenbaum G.A., Lehmann P.V. (2024). Four-Color ImmunoSpot® Assays Requiring Only 1-3 mL of Blood Permit Precise Frequency Measurements of Antigen-Specific B Cells-Secreting Immunoglobulins of All Four Classes and Subclasses. Methods Mol. Biol..

[26] Pinna D., Corti D., Jarrossay D., Sallusto F., Lanzavecchia A. (2009). Clonal dissection of the human memory B-cell repertoire following infection and vaccination. Eur. J. Immunol..

[27] Carlock M.A., Ingram J.G., Clutter E.F., Cecil N.C., Ramgopal M., Zimmerman R.K., Warren W., Kleanthous H., Ross T.M. (2019). Impact of age and pre-existing immunity on the induction of human antibody responses against influenza B viruses. Hum. Vaccines Immunother..

[28] Ecker J.W., Kirchenbaum G.A., Pierce S.R., Skarlupka A.L., Abreu R.B., Cooper R.E., Taylor-Mulneix D., Ross T.M., Sautto G.A. (2020). High-Yield Expression and Purification of Recombinant Influenza Virus Proteins from Stably-Transfected Mammalian Cell Lines. Vaccines.

[29] Köppert S., Wolf C., Becza N., Sautto G.A., Franke F., Kuerten S., Ross T.M., Lehmann P.M., Kirchenbaum G.A. (2021). Affinity Tag Coating Enables Reliable Detection of Antigen-Specific B Cells in ImmunoSpot Assays. Cells.

[30] Karulin A.Y., Katona M., Megyesi Z., Kirchenbaum G.A., Lehmann P.V. (2024). Artificial Intelligence-Based Counting Algorithm Enables Accurate and Detailed Analysis of the Broad Spectrum of Spot Morphologies Observed in Antigen-Specific B-Cell ELISPOT and FluoroSpot Assays. Methods Mol. Biol..

[31] Hanson J., Sundararaman S., Caspell R., Karacsony E., Karulin A.Y., Lehmann P.V. (2015). ELISPOT Assays in 384-Well Format: Up to 30 Data Points with One Million Cells. Cells.

[32] Dittrich M., Lehmann P.V. (2012). Statistical analysis of ELISPOT assays. Methods Mol. Biol..

[33] Xu G.J., Kula T., Xu Q., Li M.Z., Vernon S.D., Ndung’u T., Ruxrungtham K., Sanchez J., Brander C., Chung R.T. (2015). Viral immunology. Comprehensive serological profiling of human populations using a synthetic human virome. Science.

[34] Viana R., Moyo S., Amoako D.G., Tegally H., Scheepers C., Althaus C.L., Anyaneji U.J., Bester P.A., Boni M.F., Chand M. (2022). Rapid epidemic expansion of the SARS-CoV-2 Omicron variant in southern Africa. Nature.

[35] Becza N., Liu Z., Chepke J., Gao X.H., Lehmann P.V., Kirchenbaum G.A. (2024). Assessing the Affinity Spectrum of the Antigen-Specific B Cell Repertoire via ImmunoSpot®. Methods Mol. Biol..

[36] Lehmann P.V., Liu Z., Becza N., Valente A.V., Wang J., Kirchenbaum G.A. (2024). Monitoring Memory B Cells by Next-Generation ImmunoSpot® Provides Insights into Humoral Immunity that Measurements of Circulating Antibodies Do Not Reveal. Methods Mol. Biol..

[37] Baboo J., Kilbride P., Delahaye M., Milne S., Fonseca F., Blanco M., Meneghel J., Nancekievill A., Gaddum N., Morris G.J. (2019). The Impact of Varying Cooling and Thawing Rates on the Quality of Cryopreserved Human Peripheral Blood T Cells. Sci. Rep..

[38] Meneghel J., Kilbride P., Morris J.G., Fonseca F. (2019). Physical events occurring during the cryopreservation of immortalized human T cells. PLoS ONE.

