# Supplementary material for: Optimizing PBMC Cryopreservation and Utilization for ImmunoSpot® Analysis of Antigen-Specific Memory B Cells
Source: Vaccines (Basel). 2025 Jul 19;13(7):765. doi: 10.3390/vaccines13070765 (PMC12299797; doi:10.3390/vaccines13070765)
Supplement: Supplementary file 1 [file vaccines-13-00765-s001.zip › Figures and Tables.pdf]

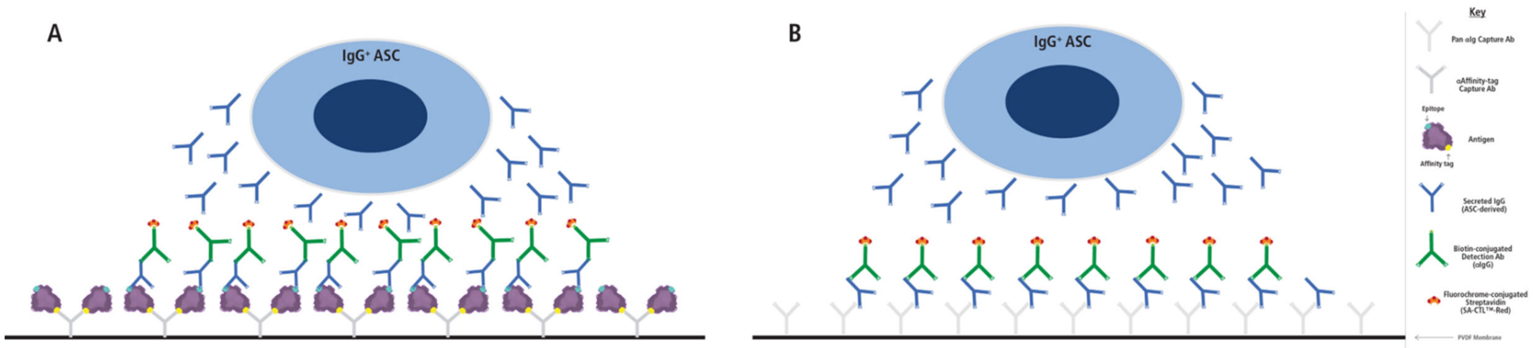

Supplemental Figure S1. B cell ImmunoSpot® assays for detection of antigen-specific or pan (total) IgG secretory footprints. A) Since many antigens cannot be efficiently absorbed directly to the assay membrane, and this undermines optimal detection of antigen-specific ASC, an alternative approach termed affinity capture coating can be leveraged to achieve high density antigen coating and maximal detection of antigen-specific ASC [24]. Specifically, instead of relying on low affinity interactions between the antigen of interest and the assay membrane, the membrane is first coated with an affinity tag (e.g. His)-specific antibody (shown in gray), followed by the tag-bearing antigen itself (in purple). In this way, low affinity absorption of the antigen to the membrane is replaced by high affinity binding, making this variant applicable for any tagged antigen. When antibody secreting B cells (ASC) are plated onto such an antigen-coated surface, the antibodies produced by the antigen-specific ASC (shown in blue), as opposed to antibodies produced by B cells that are specific for other antigens, will be captured on the membrane as a secretory footprint. B) In a pan (total) IgG detection assay the Ig produced by an ASC is captured irrespective of antigen specificity by an anti-species antibody coated onto the membrane (e.g. goat anti-human Igκ/λ, depicted in gray). In both (A) antigen-specific or (B) pan IgG-detecting ImmunoSpot® assays, plate-bound IgG secretory footprints are visualized using an anti-human IgG Fc-specific detection antibody (depicted in green) followed by deposition of a precipitating visible substrate (ELISPOT, not shown) or by selective excitation of the fluorophore-conjugated detection reagent and measurement of the resulting fluorescence using a suitable instrument (FluoroSpot, as shown).

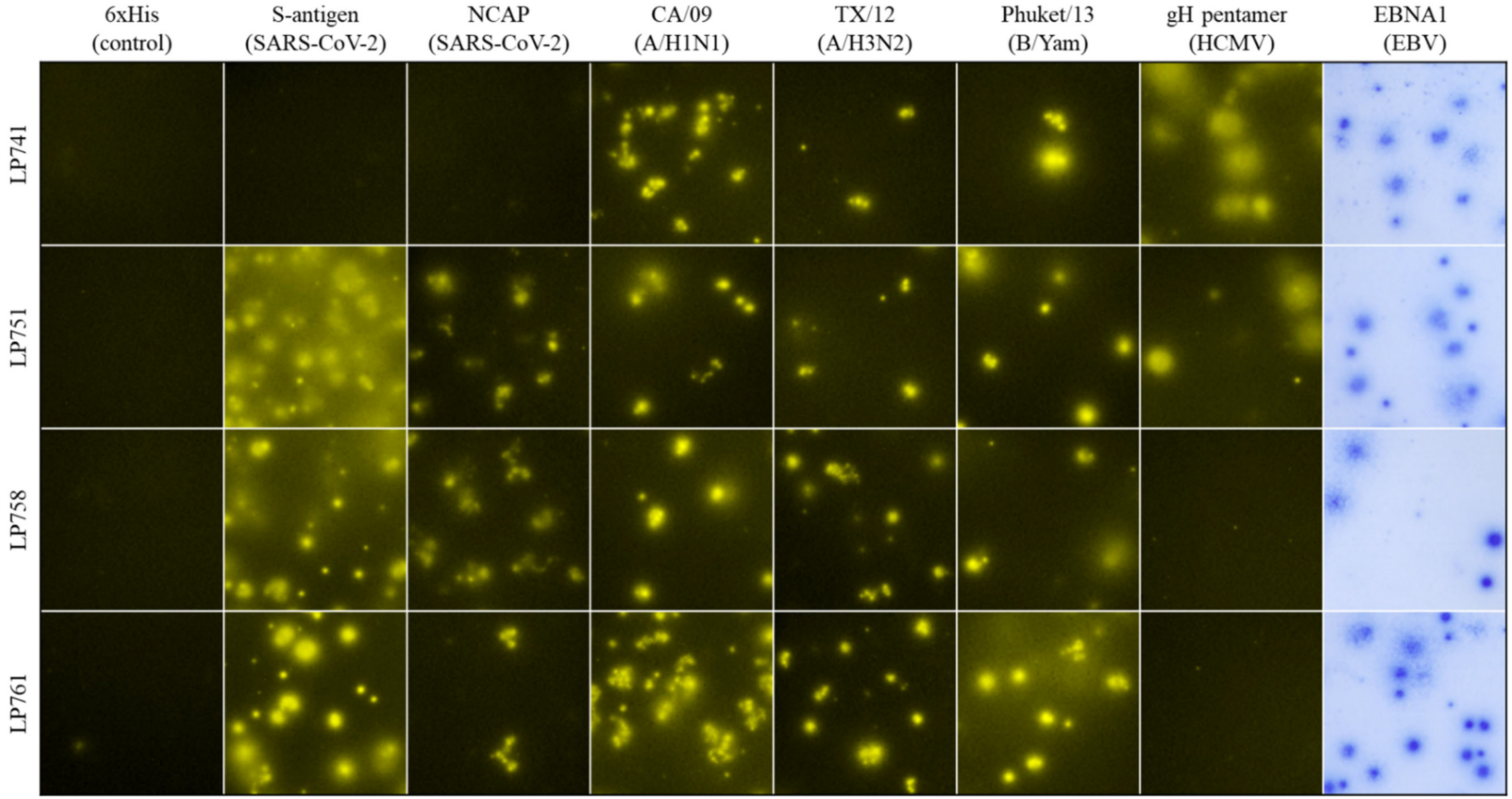

Supplemental Figure S2. B_{mem}-derived IgG^+ secretory footprints occur with diverse morphologies in ImmunoSpot® assays detecting ASC specific for different antigens. Representative spot forming units (SFUs) for the specified antigens are shown for four human subjects. Note the abundance and morphology of B_{mem}-derived IgG^+ SFU detected at 3 x 10^5 PBMC/well, and also the spectrum of morphologies occurring within the same assay well. The latter reflects the individual B cells´ different antibody productivity rates and affinity for the antigen (30). Well segments are shown with contrast enhancement to aid visualization.

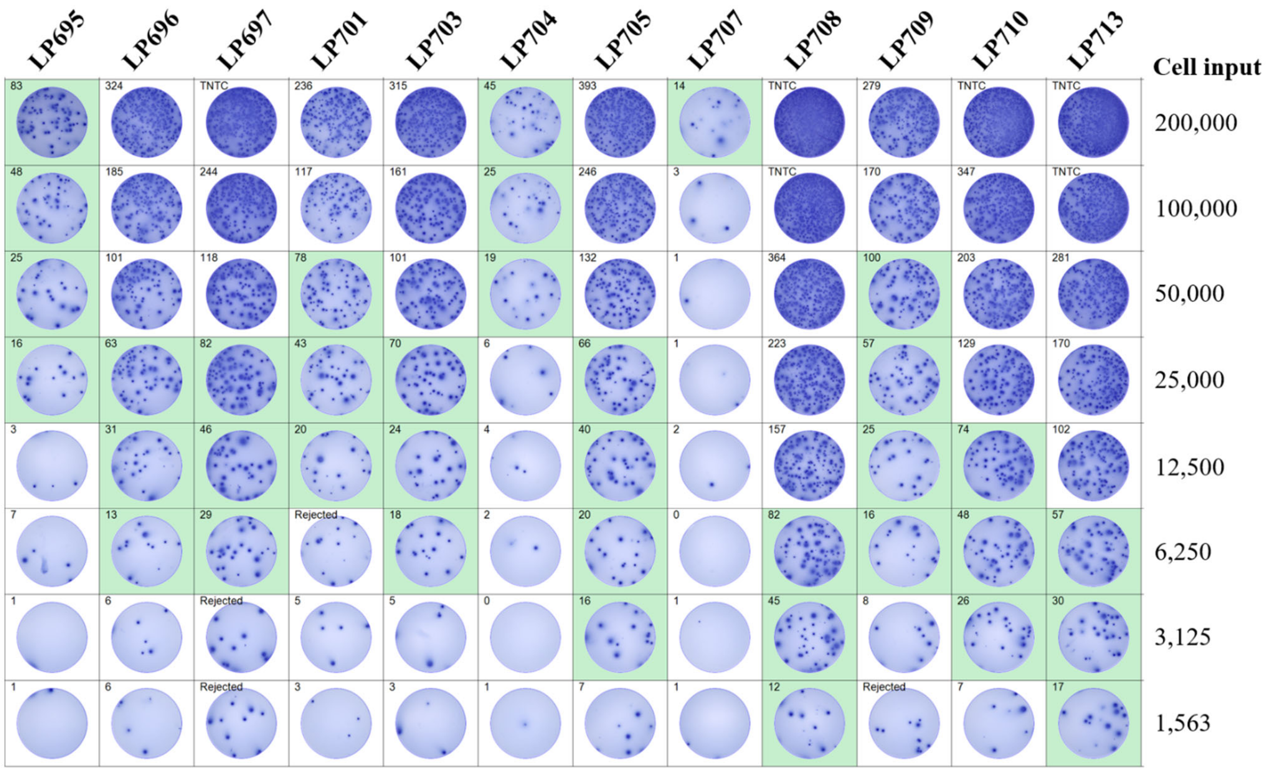

Supplemental Figure S3. Serial dilution permits reliable assessment of variable S-antigen-reactive IgG$^+$ ASC frequencies. A representative ELISPOT plate overview depicting assessment of 12 donors collected in the post-COVID era using a singlet serial dilution approach. SFU counts were enumerated using ImmunoSpot® Studio.SC software and B cell IntelliCount™ algorithms [30]. Wells yielding SFU counts within the linear range (10-100 SFU/well) are denoted with green shading and were used for frequency extrapolations (refer to Supplemental Figure S4). Notably, 11 of 12 donors possessed elevated frequencies of S-antigen-reactive IgG$^+$ ASC that yielded three or four points within the dilution range; albeit at variable cell inputs. The 8th donor (LP707) possessed a low frequency of S-antigen-reactive IgG$^+$ ASC and retesting this sample in replicate wells seeded with 2-5 x 10^5 PBMC/well would yield a more accurate frequency assessment.

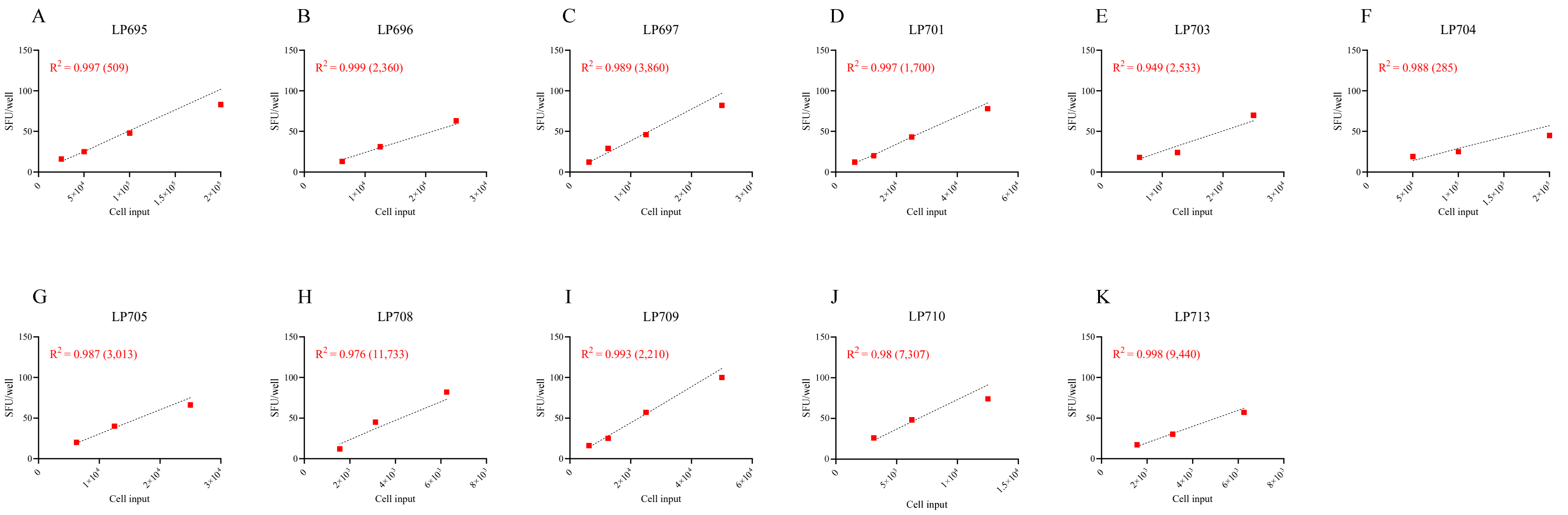

Supplemental Figure S4. Quantification of S-antigen-reactive IgG⁺ ASC using a singlet serial dilution approach. A-K) SFU counts from the indicated wells denoted in Supplemental Figure S3 for 11 of 12 test subjects were plotted on a linear scale. The dashed lines in each plot denotes the trajectory of expected SFU counts according to the calculated frequencies. R^2 values denoting the goodness of fit of the data for each donor, along with the extrapolated frequency of S-antigen-reactive IgG^+ ASC per 10^6 PBMC, are shown in the inset of each subpanel. Note: LP707 in the preceding Supplemental Figure S3 was excluded from this analysis because the test only yielded a single SFU count falling between the lower and upper bounds of 10 and 100 SFU/well, respectively. For such a test sample that yielded a low SFU count at the highest cell input tested, re-testing of the sample in which multiple replicate wells were seeded with cell input of 2-5 x 10^5 PBMC would provide a more accurate frequency assessment.

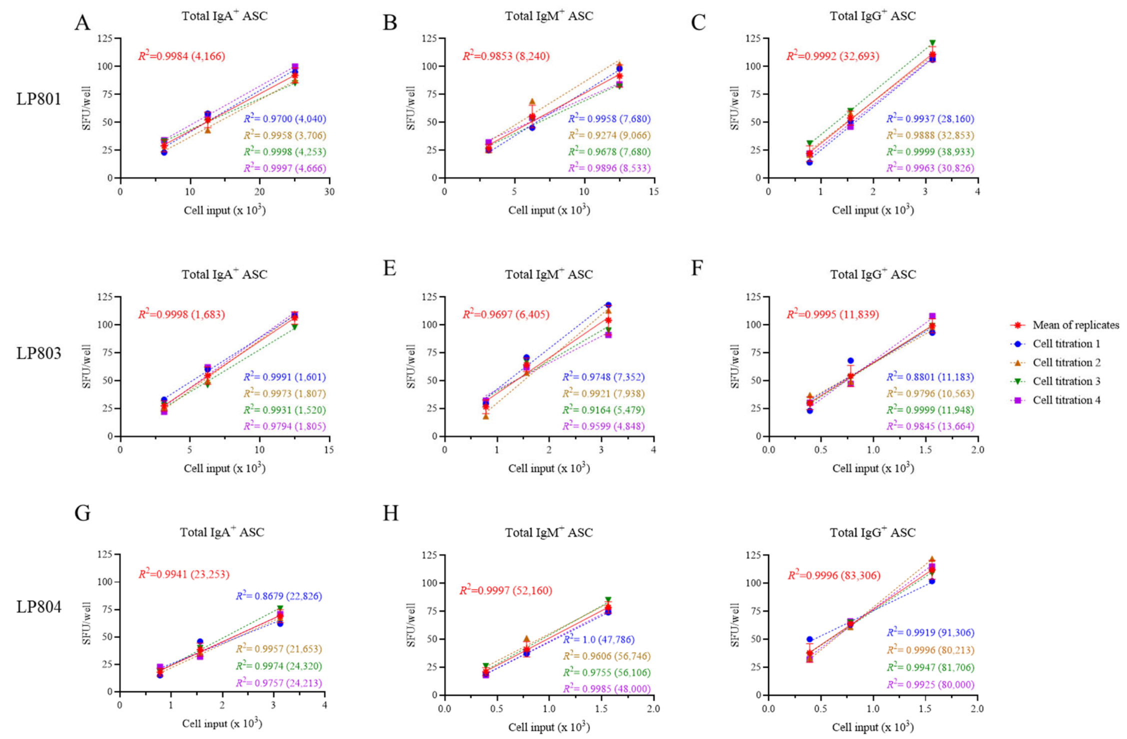

Supplemental Figure S5. Quantification of pan (total) Ig ASC using a singlet serial dilution approach is similar to that based on the mean of quadruplicates. A-I) PBMC from three healthy human subjects were subjected to in vitro polyclonal stimulation for 5 days and then tested in multiplexed FluoroSpot assays to determine the frequency of IgA+, IgM+ or IgG+ ASC using a two-fold serial dilution approach with quadruplicate measurements at each cell input. Only SFU counts occurring within the linear range of the dilution series for each donor are plotted. The mean ± SD SFU counts from the four replicate wells, along with the regression line and R^2 values, are in red. Additionally, datapoints originating from the four singlet serial dilutions, respectively, were used for linear regression analysis and the corresponding trend lines and R^2 values are denoted in the colors indicated in the figure inset. The extrapolated frequencies of ASC per 10^6 PBMC are denoted in parentheses in the corresponding colors.

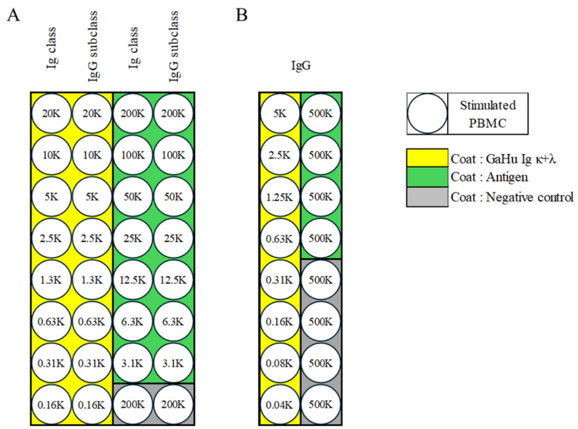

Supplemental Figure S6. Plating approach for optimal cell utilization or improving the limit of detection in B cell ImmunoSpot® assays. A) Recommended plate layout for determining the frequency of pan (total) and antigen-specific B_{mem}-derived ASC in a multiplexed FluoroSpot assay following in vitro polyclonal stimulation of PBMC using a singlet two-fold serial dilution approach. Note: performing a two-fold serial dilution starting at 2 x 10^5 PBMC for antigen-specific assays (depicted as green wells) is a reliable approach for determining the frequency of antigen-specific ASC that exist in the intermediate to high range. Similarly, unless particularly interested in the rarer Ig classes/subclasses, starting the serial dilution at 2 x 10^4 PBMC/well in multiplexed pan Ig assays (depicted as yellow wells) is sufficient. Using this approach, <1 x 10^6 PBMC following the in vitro polyclonal stimulation are required; however, inclusion of a negative antigen control (depicted as gray wells) is still advisable but requires an additional 4 x 10^5 PBMC. B) Recommended plate layout for determining the frequency of antigen-specific IgG^+ ASC that exist in the low to very low range in a single-color IgG ELISPOT assay. The detection limit of any antigen-specific assay can be progressively lowered by seeding an increasing number of replicate wells with ≤5 x 10^5 PBMC; however, cell inputs exceeding 5 x 10^5 PBMC/well are not recommended since such assay conditions can undermine the ability to discern individual secretory footprints (refer to Supplemental Figure S7). Furthermore, inclusion of negative antigen control wells (depicted as gray wells) seeded with an equivalent number of the donor's PBMC is advisable when attempting to detect rare antigen-specific ASC since these wells control for chance, non-antigen-specific secretory footprint formation; which in our experience is a rare occurrence, but if it occurs is donor intrinsic and variable.

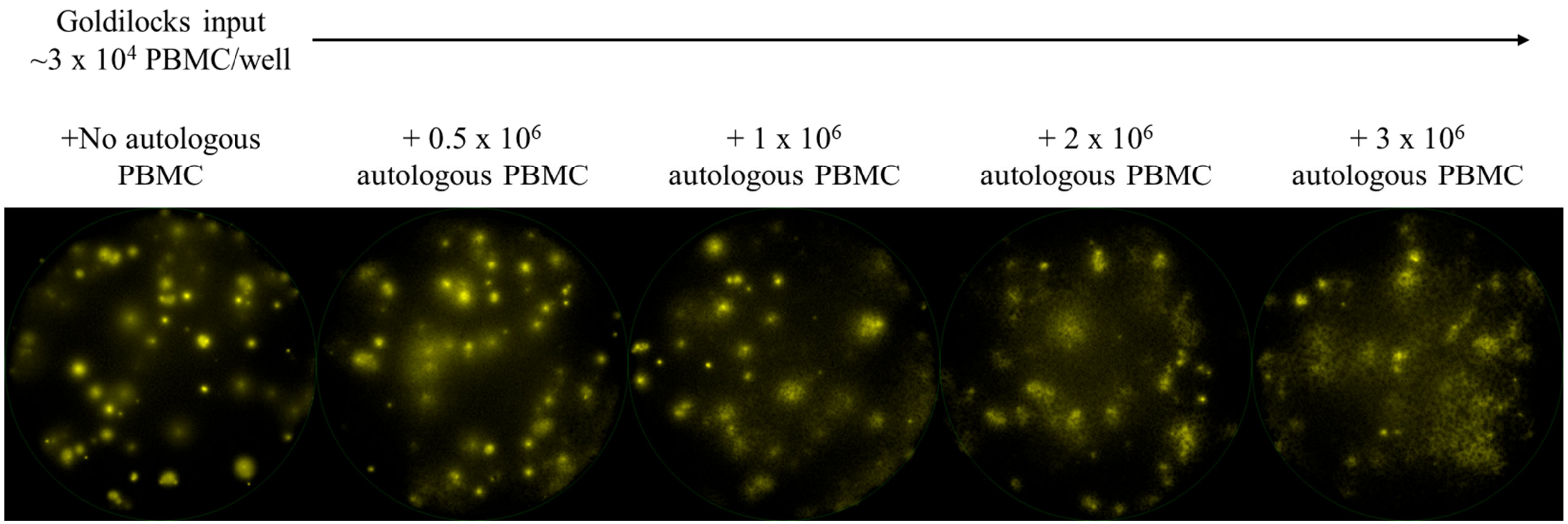

Supplemental Figure S7. PBMC inputs exceeding 5 x 10^5 per well can undermine the ability to discern individual antigen-specific secretory footprints. PBMC from a convalescent donor (LP553) with PCR-verified SARS-CoV-2 infection were polyclonally stimulated and then input into S-antigen coated wells at a "Goldilocks" input (~3 x 10^4 PBMC/well) yielding ~50 spot forming units (SFU). Representative well images depicting how cell inputs exceeding 5 x 10^5 PBMC/well can undermine optimal secretory footprint formation and reduce the ability to discern individual SFU are shown. Namely, admixing increasing numbers of unstimulated autologous PBMC lacking S-antigen-specific ASC activity (data not shown) with the donor-specific "Goldilocks" PBMC input progressively impaired the morphology and ability to resolve individual S-antigen-specific SFU.

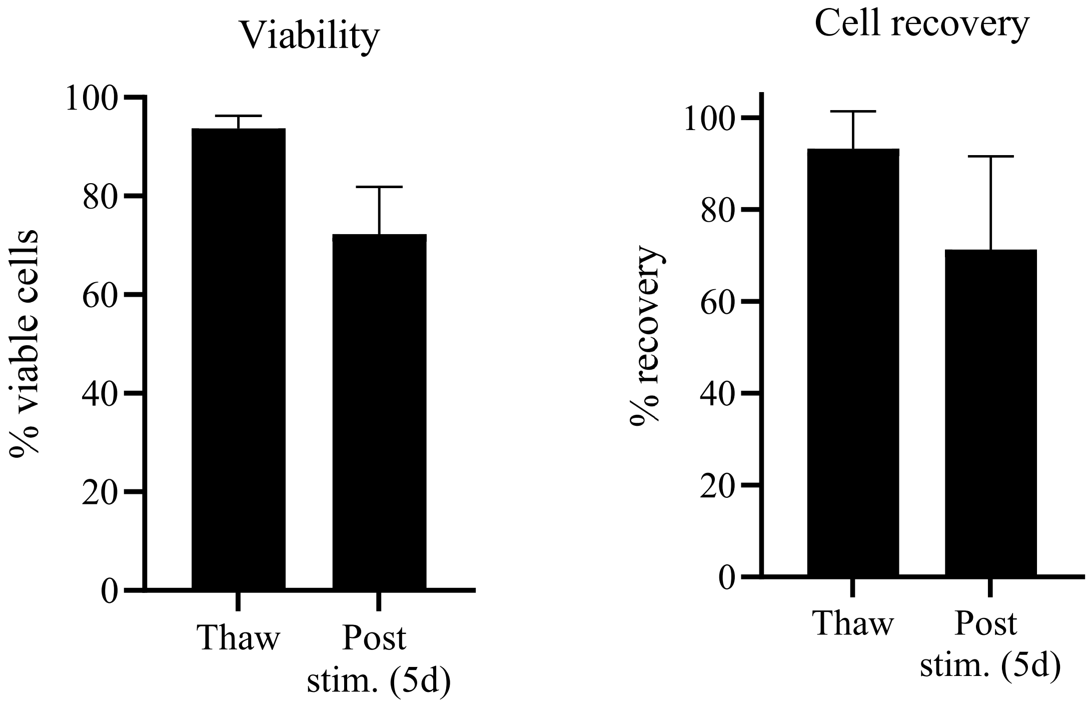

Supplemental Figure S8. PBMC viability and cell recovery following thawing and after polyclonal stimulation. Representative data from healthy human donors (n=50) depicting the viability (panel A) and cell recovery (panel B) upon thawing cryopreserved PBMC or following 5 days of in vitro polyclonal stimulation. The mean ± SD viability was 94% ± 3 upon thawing of cryopreserved PBMC and 72% ± 10 following 5 days of in vitro polyclonal stimulation. Cell recovery was 93% ± 8 upon thawing of cryopreserved PBMC and 71% ± 20 following the 5 days of polyclonal stimulation. Notably, there was more donor-to-donor variability in PBMC viability and cell recovery following the 5 days polyclonal stimulation compared to freshly thawed samples.

Supplemental Table S1. Viral antigen-specific B_{mem}-derived IgG^+ ASC frequencies vary considerably between individual donors. PBMC from healthy human subjects (n=20) were tested in ImmunoSpot® assays following in vitro polyclonal stimulation to assess B_{mem}-derived IgG^+ ASC reactivity against a panel of recombinantly expressed His-tagged antigens representing ubiquitously encountered viruses. PBMC were tested as specified in Figure 1. Spot forming unit (SFU) counts in wells input with 3×10^5 PBMC are denoted, with SFU counts >100 indicated in bold. Since quantification of wells with >125 antigen-specific SFU is generally an underestimate, see Fig. 2, we defined this as the upper limit for accurate counts.

	Viral Antigens							
Donors	S-antigen (SARS-CoV-2)	NCAP (SARS-CoV-2)	CA/09 rHA (A/H1N1)	TX/12 rHA (A/H3N2)	Phuket/13 rHA (B/Yam)	EBNA1 (EBV)	gH pentamer (HCMV)	6xHis (control)
LP724	**>125**	**>125**	**>125**	4	14	8	1	0
LP726	**>125**	34	94	12	31	21	26	0
LP727	**>125**	**>125**	57	40	34	0	3	0
LP728	85	2	16	18	5	2	0	0
LP730	57	41	50	14	31	14	28	0
LP731	**>125**	5	**>125**	50	**103**	11	0	0
LP735	**>125**	41	6	3	7	4	4	0
LP736	**>125**	14	6	5	13	11	1	0
LP738	**>125**	0	3	15	6	13	17	0
LP739	**>125**	85	29	39	22	**112**	1	0
LP740	**>125**	**118**	19	36	39	79	34	1
LP741	16	1	85	16	26	84	**116**	1
LP749	61	13	6	2	2	29	3	1
LP751	**>125**	**120**	27	28	45	67	23	0
LP756	82	17	21	14	13	28	5	0
LP757	**116**	3	**>125**	11	59	22	0	0
LP758	**>125**	**>125**	77	**>125**	30	36	1	0

LP760	**>125**	19	88	52	50	27	**119**	0
LP761	**>125**	27	**>125**	50	70	**109**	5	1
LP769	**>125**	99	83	42	29	1	4	0

Supplemental Table S2. Day-to-day variability of S-antigen-reactive IgG+ SFU counts. Three aliquots of cryopreserved PBMC obtained from a single blood draw of the specified S-antigen-primed donors were each thawed, on different days, by a single investigator, and then subjected to polyclonal stimulation with Human B-Poly-S. For all three independent test runs, the cells were washed, counted, and then seeded in a single well 1+1 serial dilution series, starting at 3 x 10^5 PBMC/well for the S-antigen, and 3 x 10^4 PBMC/well for the pan IgG detecting ImmunoSpot® assays. The frequencies for S-antigen-reactive and pan IgG+ ASC within all PBMC were calculated using three or more points in the linear range of the respective assays. The results are presented as the percentage of S-antigen-reactive IgG+ ASC among all (pan) IgG+ ASC present in the test sample(s) for each donor in the three independent tests. The mean, standard deviation (SD) and coefficient of variation (CV, expressed as a percentage) for the results obtained in the three tests are shown to the right.

	Test 1	Test 2	Test 3	Mean	± SD	% CV
LP671	3.2%	3.3%	3.6%	3.4%	0.2%	6.6%
LP672	0.2%	0.2%	0.2%	0.2%	0.0%	5.5%
LP673	1.1%	1.2%	0.9%	1.1%	0.2%	15.4%
LP676	5.4%	4.8%	4.9%	5.0%	0.3%	5.8%
LP677	0.8%	0.8%	1.2%	0.9%	0.2%	22.8%
LP678	0.3%	0.3%	0.2%	0.3%	0.1%	18.2%
LP683	0.8%	0.7%	0.8%	0.7%	0.0%	4.6%
LP690	10.3%	11.1%	9.2%	10.2%	1.0%	9.5%

Supplemental Table S3. Variable frequencies of ASC producing the different Ig classes and IgG subclasses following polyclonal stimulation of PBMC. Following in vitro polyclonal stimulation with B-Poly-S (R848+rIL-2) for 5 days, the frequency of antibody-secreting cells (ASCs) producing the four Ig classes (IgM/IgA/IgG/IgE) or four IgG subclasses (IgG1/IgG2/IgG3/IgG4) was determined in multiplexed FluoroSpot assays using a two-fold serial dilution strategy. SFU counts occurring within the linear range for each Ig class or IgG subclass were used to calculate the frequency of pan (total) ASC, irrespective of their antigen specificity, and are expressed per 2 x 10^5 PBMC. Mean ± SD frequency (SFU per 2 x 10^5 PBMC) of Ig class- and IgG subclass-producing ASC illustrates their variable abundance amongst a representative donor cohort (n=28). N/A, not applicable; ND, not detected.

	Ig class QCF				IgG subclass QCF			
Donor	IgM	IgA	IgG	IgE	IgG1	IgG2	IgG3	IgG4
LP315	3,347	5,507	15,573	ND	11,787	1,300	891	2,208
LP326	2,020	1,893	21,120	ND	15,093	1,140	2,490	102
LP353	19,947	4,853	11,467	ND	6,667	1,583	1,003	37
LP409	11,093	1,880	24,480	ND	16,320	783	1,283	92
LP418	6,053	3,067	23,360	ND	12,213	970	1,387	128
LP424	2,750	865	6,470	ND	2,190	716	477	31
LP426	2,400	4,153	24,107	ND	10,080	1,977	2,203	326
LP432	14,773	3,867	24,533	ND	14,080	2,030	1,890	77
LP454	21,013	5,387	22,773	ND	14,400	690	864	1,405
LP456	3,045	2,020	7,227	ND	4,747	1,130	555	102
LP469	6,500	7,160	38,293	ND	19,040	2,480	1,590	382
LP472	8,256	5,093	29,920	ND	17,920	1,420	2,640	300
LP526	13,280	6,307	27,413	ND	23,467	1,505	2,467	241
LP528	7,173	3,360	20,213	ND	8,480	1,280	1,680	725
LP534	24,587	6,160	23,787	ND	16,320	2,417	1,713	299
LP542	15,147	7,227	16,747	ND	11,520	1,583	1,127	13
LP543	13,707	3,647	30,400	ND	19,467	1,533	1,180	350
LP547	10,933	469	18,453	ND	13,387	1,003	1,700	218
LP553	947	827	14,293	ND	7,733	948	635	114

LP555	34,347	7,120	26,667	ND	23,147	1,047	967	411
LP557	19,947	8,187	27,840	ND	14,827	2,633	703	318
LP560	3,585	2,073	14,453	ND	5,493	1,656	1,380	66
LP561	4,200	4,413	32,320	ND	17,707	920	2,100	522
LP564	15,840	5,560	39,360	ND	18,613	3,177	2,490	448
LP565	14,133	1,657	12,907	ND	5,920	760	873	122
LP566	28,373	9,173	28,640	ND	20,853	1,993	1,217	414
LP568	7,773	5,800	21,227	ND	10,027	973	807	362
LP569	5,360	2,360	21,067	ND	10,027	1,103	1,023	306
Mean	11,447	4,289	22,325	N/A	13,269	1,455	1,405	361
±SD	8,648	2,377	8,192	N/A	5,682	642	644	456

Supplemental Table S4. SARS-CoV-2 Spike and Nucleocapsid antigen-specific IgG+ ASC are detectable only in antigen-exposed individuals. To illustrate the diagnostic specificity for detecting underlying memory B cell reactivity against SARS-CoV-2 Spike (S-antigen) and Nucleocapsid (NCAP), cryopreserved PBMC originating from the three specified cohorts were evaluated for IgG^+ ASC reactivity in B cell ImmunoSpot® assays. Polyclonally stimulated PBMC were plated at 1 or 2 x 10^5 cells/well in S-antigen or NCAP assays, respectively. Donors yielding a SFU count exceeding the upper limit of quantification were assigned a value of >125 SFU.

Group	Samples	S-antigen	NCAP
Pre-COVID	LP377	0	2
	LP418	0	0
	LP424	0	0
	LP432	1	0
	LP453	1	0
	LP454	0	0
	LP332	0	0
	LP341	0	0
Post-Vax 2 (6-month)	CS134	67	0
	CS930	29	0
	CS512	>125	1
	CS067	28	0
	CS059	59	0
	CS131	35	0
	CS116	>125	0
	CS076	>125	0
PCR-verified	LP526	>125	>125
	LP528	17	7
	LP553	>125	98

	LP555	114	62
	LP561	>125	>125
	LP565	>125	19
	LP568	93	>125
	LP569	41	9

Supplemental Table S5. Lowering the detection limit of SARS-CoV-2 NCAP-specific B_{mem}-derived IgG^+ ASC. PBMC samples (n=10) collected in 2022 (between May and October, refer to supplemental excel file for additional details) were polyclonally stimulated and then input into single-color ELISPOT assays to detect pan (total) IgG^+ ASC or those with reactivity against SARS-CoV-2 S-antigen, NCAP or irrelevant antigen controls, respectively. PBMC were tested using a singlet two-fold serial dilution approach starting at 5 x 10^3 or 2 x 10^5 PBMC/well for the pan IgG and S-antigen assays, respectively. For the NCAP assay, PBMC samples were tested using a singlet serial dilution approach starting at 2 x 10^5 PBMC/well or at 5 x 10^5 cells/well in four replicate wells. For the irrelevant antigen controls (6xHis or BSA), PBMC were tested at 5 x 10^5 PBMC/well in four replicates. Spot forming unit (SFU) counts for pan IgG^+ or S-antigen-reactive IgG^+ ASC were extrapolated to 2 x 10^5 PBMC based on frequencies determined using counts occurring in the linear range of the respective assays. SFU counts from the NCAP-coated wells seeded with 2 x 10^5 PBMC, along with one replicate well coated with NCAP or the irrelevant antigen controls seeded with 5 x 10^5 PBMC, are shown for each donor. Moreover, the cumulative SFU count from four replicate NCAP-coated wells seeded with 5 x 10^5 PBMC, or with irrelevant antigen controls, are shown for each donor. The cell incubation period for these ELISPOT assays was 5 h to reduce background membrane staining and avoid crowding of secretory footprints owing to enzymatic amplification.

	SARS-CoV-2								
	FL Spike	NCAP			6xHis		BSA		Pan IgG
Donor	200K[a]	200K	500K	Cumulative[b]	500K	Cumulative[b]	500K	Cumulative[b]	200K
LP695	97	1	3	9	2	7	0	0	13,920
LP696	394	11	25	108	0	0	0	1	15,680
LP697	692	0	0	0	0	0	0	0	12,220
LP701	310	3	9	35	1	2	0	0	13,620
LP703	449	14	41	172	0	0	1	2	6,910
LP704	42	16	42	178	0	0	0	0	8,350
LP705	624	19	40	168	0	0	0	0	22,720
LP708	1,877	30	95	338	0	0	0	0	13,580
LP709	383	5	14	59	0	1	0	0	13,216

LP710	1,253	3	12	49	0	0	0	0	20,320

[a] denoted SFU values were extrapolated from data generated using a singlet serial dilution testing approach

[b] sum of 4 replicate wells tested at 500K PBMC
